# Supplementary material for: Responses of Bilevel Ventilators to Unintentional Leak: A Bench Study
Source: Healthcare (Basel). 2022 Nov 30;10(12):2416. doi: 10.3390/healthcare10122416 (PMC9777664; doi:10.3390/healthcare10122416)
Supplement: Supplementary file 1 [file healthcare-10-02416-s001.zip › healthcare-2014659-supplementary.pdf]

Supplementary

	Pulmonary model	Expiratory trigger setting	Measured %Qflow	Pressure rise time setting	Measured rise time
DreamStation BiPAP AVAPS	COPD	Autotrak	29%	1	336 ± 4 ms
	OHS	Autotrak	15%		
	NMD	Autotrak	18%		
Lumis 150 VPAP ST	COPD	High	37%	200 ms	276 ± 2 ms
	OHS	Low	15%		
	NMD	Medium	22%		
Vendom 40	COPD	50%	42%	2	294 ± 26 ms
	OHS	25%	16%		
	NMD	25%	18%		

Table S1: Expiratory trigger sensitivities (corresponding %Qflow) and pressure rise time (corresponding measured rise time) for each device and respiratory model. %Qflow: percentage of the peak flow during the inspiratory phase.

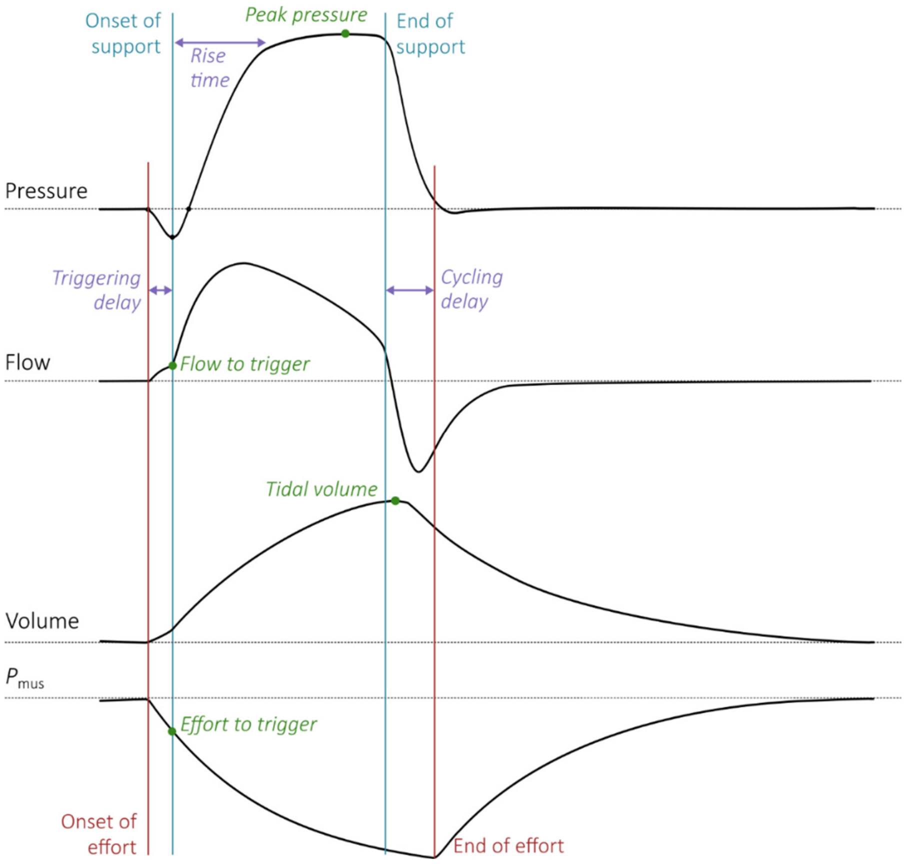

Figure S1. Description of how ventilator performance was assessed. The onset of pressure support allows measurement of the triggering delay and the flow to trigger, while the return to expiratory pressure allows measurement of the cycling delay. The maximal delivered pressure, the pressure rise time and the tidal volume were measured from the ASL-5000 airway pressure and piston volume. Pmus: muscular pressure.

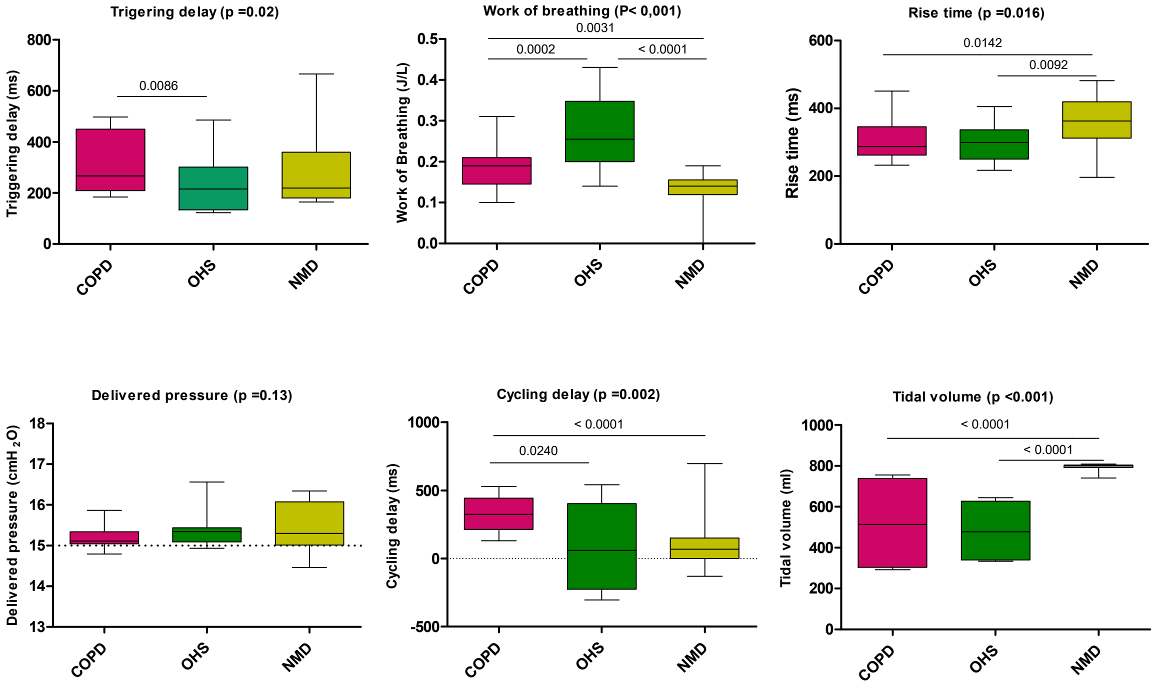

Figure S2. Consolidated data integrating the 3 ventilators with continuous leakage according to the 3 respiratory models with and without flow limitation on triggering delays, work of breathing, pressure rise time, delivered pressure, cycling delay and tidal volume

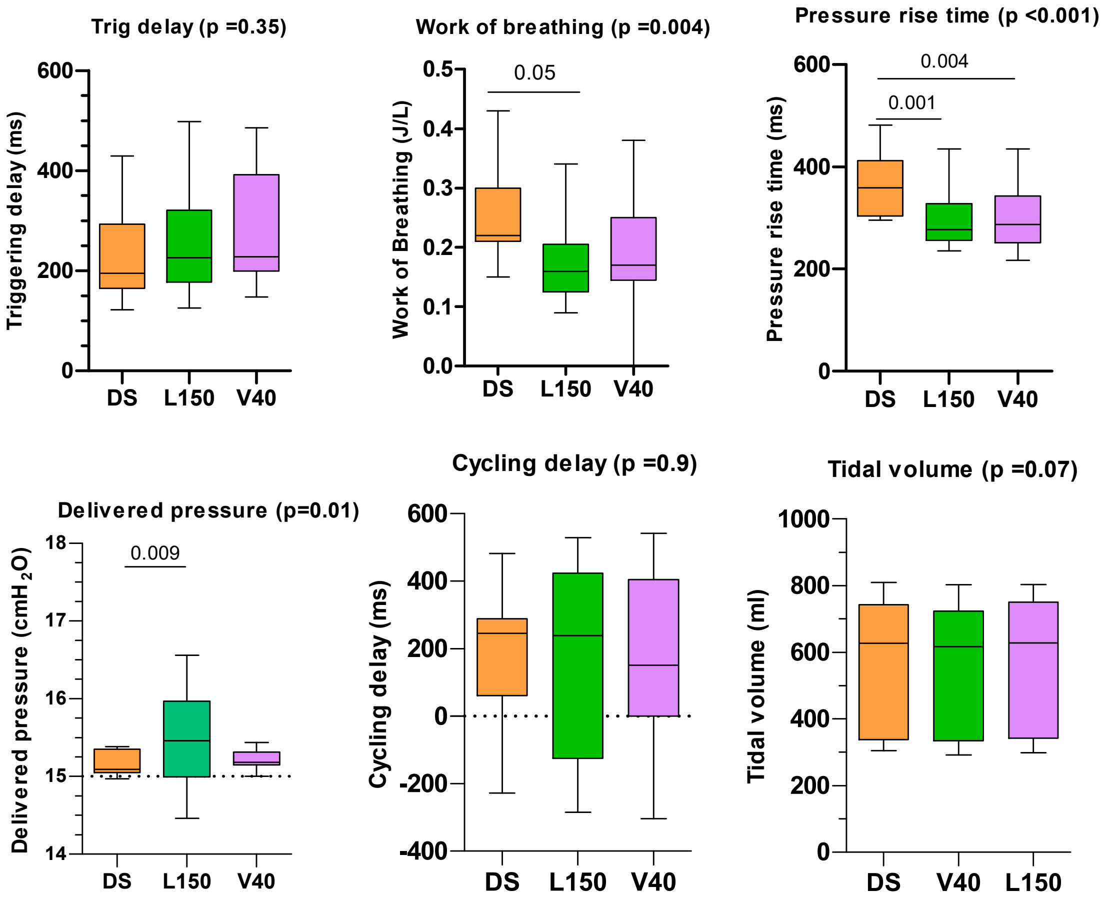

Figure S3. Consolidated data integrating the 3 respiratory models with continuous leakage according to the 3 respiratory ventilators with and without flow limmitation on triggering delays, work of breathing, pressure rise time, delivered pressure, cycling delay and tidal volume.
